# Cognitive Enhancement Through Music Education: Affective Pathways to Executive Function Improvement in Musicians

**DOI:** 10.3390/brainsci16020161

**Published:** 2026-01-30

**Authors:** Evgenia Gkintoni, Helen Kanellopoulou, Christos Pouris, Stephanos P. Vassilopoulos, Georgios Nikolaou, Constantinos Halkiopoulos

**Affiliations:** 1 University General Hospital of Patras, 26504 Patras, Greece; 2 Department of Educational Sciences and Social Work, University of Patras, 26504 Patras, Greece; stephanosv@upatras.gr (S.P.V.); gnikolaou@upatras.gr (G.N.); 3 Department of Management Science and Technology, University of Patras, 26504 Patras, Greece; up1103232@ac.upatras.gr (H.K.); up1111321@upatras.gr (C.P.); halkion@upatras.gr (C.H.); 4 National Conservatory-Annex of Vrilissia, 15235 Athens, Greece

**Keywords:** executive function, Stroop test, music education, positive affect, state anxiety, path analysis, mediation, cognitive enhancement, educational neuroscience, neuropsychology, broaden-and-build theory, processing efficiency

## Abstract

*Background*/*Objectives*: This pilot study employed a quasi-experimental, single-group, pre-post design to examine the acute effects of single music lessons on executive function and to explore whether affective changes are associated with cognitive improvement in trained musicians. Drawing on Fredrickson’s broaden-and-build theory and Eysenck’s processing efficiency theory, we hypothesized that changes in positive affect and state anxiety would be statistically associated with cognitive outcomes. *Methods*: Using purposive sampling, 60 musicians (34 female, 26 male; Mage = 26.0, SD = 9.8; range: 16–58 years) completed assessments before and after a 45–60 min instrumental lesson (guitar, *n* = 20; violin, *n* = 20; piano, *n* = 20). Executive function was measured using the Stroop Color-Word Test (Golden version, Greek-validated). Affective states were assessed using the Positive and Negative Affect Schedule (PANAS; 20 items) and State-Trait Anxiety Inventory-State (STAI-S; 20 items). Data were analyzed using paired *t*-tests, Pearson correlations, path analysis, and bootstrap mediation analysis (5000 resamples). *Results*: Music lessons were associated with improved executive function (Stroop interference: d = 0.59, *p* < 0.001), increased positive affect (d = 1.87, *p* < 0.001), and reduced negative affect (d = −2.34, *p* < 0.001) and state anxiety (d = −2.64, *p* < 0.001). Path analysis demonstrated excellent model fit (CFI = 1.00; RMSEA = 0.00), with affective changes associated with 61.3% of the total effect on cognitive improvement. *Conclusions*: Single music lessons were associated with both cognitive and affective benefits, with affective changes statistically linked to cognitive outcomes. As a pilot study, these exploratory findings require replication using controlled designs before generalization. Future research should incorporate neuroimaging methods and cross-cultural validation.

## 1. Introduction

Music is one area of human learning where many brain systems are used at the same time—auditory processing, motor skills, paying attention, remembering, controlling emotions, and social awareness all combine when we engage with music as do very few other areas of experience [1,2,3]. Research over the last 20 years has shown that music training enhances certain cognitive abilities; specifically, it enhances the development of executive functions: higher-order cognitive processes that support goal-directed behaviors, thinking flexibly and regulating oneself [4,5,6]. Although there is an increasing appreciation for the cognitive advantages of music education, the psychological and neuro-biological pathways that provide the basis for the cognitive advantages provided by music have been insufficiently identified, especially the affective pathways that link music and cognition [7,8,9,10,11,12].

### 1.1. The Stroop Effect and Executive Function

The Stroop Color-Word Test is an extremely popular assessment of the neuropsychological constructs of executive function—specifically, it assesses Selective Attention (the ability to focus on relevant stimuli while ignoring irrelevant stimuli), Cognitive Flexibility (the ability to switch between different mental tasks) and Response Inhibition (the ability to suppress inappropriate responses). The Stroop effect has been shown to be a highly sensitive measure of these constructs [13,14].

John Ridley Stroop originally reported the interference phenomenon that underlies the Stroop effect in 1935. He found that when individuals are required to name the ink color of words printed in different colors (e.g., the word “red” written in blue ink), they require suppressing their automatic reading response to access the appropriate color-naming response [15]. What appears to be such a simple task reveals fundamental principles of cognitive control that have captured researchers’ interest for nearly a century [16,17,18,19,20].

Brain imaging studies have consistently demonstrated that performance on the Stroop task activates two regions of the brain that play a critical role in conflict monitoring, error detection and top-down cognitive control—the anterior cingulate cortex and the dorsolateral prefrontal cortex [21,22]. Furthermore, the degree of Stroop interference and how well it can be resolved reflects an individual’s executive function capacity. Therefore, the Stroop task is a particularly useful tool for studying the effects of various forms of cognitive enhancement [23].

Recent advancements in EEG technology have also provided additional insight into the timing of Stroop interference—that is, the time course of conflict detection and resolution—and have identified specific neural patterns that occur at particular times during the process [24,25]. These objective neurophysiologic markers of executive function provide a complementary approach to the assessment of executive function and have the potential to reveal the underlying brain mechanisms responsible for cognitive control [26,27,28,29,30,31,32,33].

### 1.2. Musical Training and Cognitive Enhancement

Cognitive neuroscience researchers are increasingly interested in how musical training contributes to enhanced executive function. Research has shown that musicians consistently perform better than non-musicians on various assessments of attention, working memory, and cognitive flexibility. Additionally, long-term studies indicate that some of the advantages seen in musicians result from their musical training, rather than existing prior to the start of their training [34,35,36].

In addition to the research demonstrating that musicians are superior to non-musicians in terms of cognitive abilities, researchers have also developed a new tool known as the “Musical Stroop” paradigm. This new tool assesses how much interference occurs when musicians are required to suppress their natural tendency to read musical notes (the automatic process) while completing a task; this study found that the amount of interference increased proportionally to the number of years of musical training (similar to the way in which interference increases as one learns to suppress automatic word reading in the classic Stroop task) [12,37,38,39,40,41].

Research using neuroimaging techniques is providing substantial evidence that training on music can lead to neurological changes or music-induced neuroplasticity. Longitudinal studies have followed children who received music training over time and have observed structural changes in both the child’s auditory cortex and motor cortex as well as an enlargement of the corpus callosum facilitating communication between the two hemispheres of the brain and the formation of new neural connections in the prefrontal areas of the brain responsible for executive function [42,43]. These findings are consistent with the principles outlined in educational neuroscience that emphasize that extended periods of learning will alter the structure and function of the brain in ways that go beyond what was originally learned [44]. Furthermore, research on the brain’s ability to undergo experience dependent changes indicates that music education can provide a powerful means to support cognitive development, especially during periods of critical neural growth and development [45,46,47,48,49,50,51].

### 1.3. Affective Pathways in Music-Related Cognitive Enhancement

There is substantial evidence showing that musical education enhances cognitive abilities; however, recent studies suggest that the emotional aspects of music could also be involved as a mediator for the cognitive improvement produced by music. In other words, music produces reliable emotional responses—from subtle variations in mood when listening to background music to powerful emotional experiences of attending concerts—and these emotional modifications can produce cognitive consequences [52,53]. Neuroscience studies on music and emotion demonstrate that music activates structures of the brain’s limbic system (amygdala, nucleus accumbens, and orbito-frontal cortex), which are connected to the prefrontal executive systems [24,54,55,56,57,58,59,60,61,62,63,64].

The broaden-and-build theory of positive emotions offers a theoretical model to understand how the positive emotions produced by music could positively influence cognitive performance [65]. This model indicates that positive emotions increase the breadth of attention and cognition, which increases the variety of thought and behaviors that are considered and builds lasting personal resources. On the neural level, positive affect is linked with greater dopamine release in the mesocorticolimbic pathways that regulate prefrontal function and thereby promote cognitive flexibility and creative problem solving [66,67]. Music’s ability to provide a reliable means of producing positive emotional states may therefore establish neurochemical conditions that support enhanced executive functions [11,68,69,70,71,72,73,74].

On the contrary, anxiety and negative affect restrict the scope of attention and diminish executive function via their impacts upon working memory capacity. Processing efficiency theory proposes that anxiety consumes cognitive resources by inducing worry and monitoring threats, and as such leaves fewer cognitive resources available for relevant-task processing [75,76]. Music’s documented anxiolytic properties—illustrated in its use in preparing patients for surgery to treating disorders of anxiety—may improve cognition by liberating working memory resources that would otherwise be employed to ruminative thinking due to anxiety. Recent developments in emotion recognition using EEG offer real-time assessments of emotional states while engaged with music, and thereby allow for the study of the temporal dynamics underlying music’s modulation of emotional processing [24,77]. Such neurophysiological methods promise to provide useful tools for studying the interplay among musical experience, emotional response and cognitive function at each moment [78,79,80,81,82,83].

### 1.4. Mental Health, Educational Neuroscience, and Applied Implications

The inclusion of mental health aspects in educational practice is becoming a high-priority issue; which has significant impacts upon the learning outcome of students [84,85]. Anxiety disorders have been found to impact a large portion of students, and research demonstrates these disorders are consistent with poor academic performance, decreased cognitive flexibility, and lower learning outcomes [86]. The understanding that emotional well-being and cognitive function are neurologically connected (and therefore cannot be separated) has increased interest in interventions that concurrently treat both [87,88,89,90,91,92].

Music education is uniquely positioned at the intersection of cognition and emotion. While cognitive training programs are designed to train or improve specific mental processes independently of emotional experience, music engagement involves emotional experience, social interaction, and personal meaning-making with the opportunity for skill improvement [93,94] and therefore explains the potential of music education to support cognitive enhancement as well as social-emotional development, self-esteem, and mental health outcomes. Educational neuroscience continues to recognize that effective learning involves consideration of affective and cognitive processes, and that interventions that engage multiple brain systems can yield greater, longer-lasting outcomes than those that are narrowly focused [44,95,96,97,98].

The emergence of artificial intelligence and adaptive learning technologies offers new opportunities to optimize music education. Cognitive neuropsychology-informed personalized learning systems may be used to create musical instruction tailored to the individual learner profile, including adjusting difficulty, repertoire, and instructional methods based on real-time assessments of cognitive and affective states [95]. Deep learning algorithms capable of identifying emotional responses from facial expressions, physiological signals, and behavioral patterns enable dynamically adjusted music instruction that optimizes positive engagement and minimizes frustration and anxiety [77]. The concept of digital twin cognition allows for a framework for precision music education that incorporates all aspects of individual differences in learning [99,100].

### 1.5. Cultural Context and Cross-Cultural Considerations

The impact on brain and behavior of music and its influence on emotional and cognitive responses are directly tied to the culture or cultural environment in which they take place. Cultures around the world have different scales, rhythmic patterns, instrumentation, purposes for use in social settings, and aesthetic values, which will likely affect how musical instruction influences brain function and behavior [101]. As a result of this cultural significance (in Greece), the educational system and conservatories in Greece carry a high level of prestige and provide both personal and cultural enrichment [102,103,104].

Cultural neuroscience studies have shown that cultural practices influence how the brain organizes in domain-specific ways, and thus, the cognitive changes associated with a variety of training experiences are also likely influenced by cultural context [101]. In addition, multisensory learning research has shown that mechanisms of neuroplasticity differ across cultural and linguistic backgrounds, indicating that the pathways by which music can enhance cognition will vary accordingly [101].

While there have been many studies documenting universals in music perception and emotional response to music, it is possible that the specific cognitive enhancements associated with music education are modified by cultural factors, including the social implications of musical achievement; the pedagogy used to teach music; and the larger educational and values systems within which music education takes place [105,106,107,108,109,110,111].

The above considerations suggest that a cross-cultural perspective would be beneficial for the study of music cognition and that music education research should employ culturally informed perspectives. This study is one of many being conducted internationally as part of the broader effort to understand music cognition across diverse cultural environments; however, results may require replication and extension across various cultural contexts [112,113,114,115,116,117,118,119].

### 1.6. Study Objectives and Hypotheses

The present study addresses critical gaps in the literature by simultaneously examining cognitive performance, positive and negative affect, and state anxiety before and after music lessons, employing validated instruments sensitive to acute changes. This multi-measure approach enables investigation of the relationships among affective and cognitive changes that would be invisible to studies examining either domain in isolation.

General Objective: To examine the acute cognitive and affective effects of single music lessons in trained adult musicians and to explore whether affective changes are associated with cognitive outcomes.

Specific Objectives: 1. To assess changes in executive function (Stroop interference) following a single music lesson. 2. To evaluate changes in positive affect, negative affect, and state anxiety following a single music lesson. 3. To explore whether changes in affective states are associated with changes in executive function. 4. To test statistical mediation models examining affective variables as potential pathways linking music lessons to cognitive outcomes.

The following research questions (RQ) and corresponding hypotheses (H) guide this investigation:

**[RQ1]**  What are the acute effects of music lessons on executive function as measured by Stroop test performance?

**[RQ2]**  What are the acute effects of music lessons on affective states, including positive affect, negative affect, and state anxiety?

**[H1]**  *Based on prior evidence for music’s cognitive and emotional benefits, we hypothesize that music lessons will significantly improve Stroop performance while increasing positive affect and reducing negative affect and anxiety.*

**[RQ3]**  What is the relationship between affective changes and cognitive improvement following music lessons?

**[H2]**  *Consistent with theories linking positive affect to cognitive flexibility and anxiety to executive impairment, we hypothesize that increases in positive affect and reductions in anxiety will correlate positively with cognitive improvement.*

**[RQ4]**  Do affective changes mediate the relationship between music lessons and cognitive enhancement?

**[H3]**  *Drawing on the broaden-and-build theory and processing efficiency theory, we hypothesize that affective changes—particularly positive affect enhancement and anxiety reduction—will be statistically associated with cognitive improvement in mediation models, consistent with theoretical predictions of affective pathways.*

**[RQ5]**  Are there individual differences in cognitive and affective outcomes based on demographic characteristics or instrument type?

This final question is exploratory, as the literature provides insufficient basis for directional hypotheses regarding gender, age, or instrument-specific effects.

### 1.7. Operational Definitions

For clarity, key constructs are operationally defined as follows:

- Music education refers to structured instrumental instruction delivered by qualified teachers in individual lesson format, lasting 45–60 min, involving technical exercises, repertoire practice, and musical interpretation.
- Cognitive enhancement is operationalized as improvement in executive function, specifically measured as change in Stroop interference scores (with higher scores indicating better inhibitory control) from pre- to post-lesson assessment.
- Affective pathways refers to the statistical associations between changes in emotional states (positive affect, negative affect, state anxiety) and changes in cognitive performance, tested through correlation and mediation analyses. This term denotes statistical relationships rather than confirmed causal mechanisms.

## 2. Materials and Methods

### 2.1. Research Design

This pilot study employed a quasi-experimental, single-group, pre-post design without a control condition to examine the acute effects of music lessons on cognitive and affective outcomes. As an exploratory investigation, this study aimed to generate preliminary evidence regarding affective pathways in music-related cognitive changes and to inform the design of future controlled trials. The independent variable was participation in a single music lesson (within-subjects factor: pre-lesson vs. post-lesson). The dependent variables were: (a) executive function, measured by Stroop interference scores; (b) positive affect and negative affect, measured by the PANAS; and (c) state anxiety, measured by the STAI-S. Participants completed identical assessment batteries immediately before and after their standard music lesson. This design enables detection of within-person changes but does not permit causal inference or generalization to broader populations due to the absence of randomization and control conditions.

### 2.2. Participants

Sixty musicians (34 female, 26 male) were recruited from music conservatories and private music schools in Greece. Inclusion criteria required: (1) age ≥ 16 years; (2) currently enrolled in instrumental music lessons; (3) minimum one year of prior music training; (4) no current psychiatric diagnosis or psychoactive medication use. Participants were stratified by instrument type with equal representation of guitar (*n* = 20), violin (*n* = 20), and piano (*n* = 20). Participants under 18 years of age (*n* = 4) provided written assent, and their parents or legal guardians provided written informed consent in accordance with institutional ethics requirements. Adult participants (*n* = 56) provided written informed consent directly. Sample size was determined a priori using G*Power 3.1.9.7. For a paired-samples *t*-test detecting a medium effect (d = 0.50) with α = 0.05 and power = 0.80, the required sample was *n* = 54. We recruited 60 participants to allow for potential incomplete data, yielding actual power of 0.85 for the obtained effect size (d = 0.59). The broad age range (16–58 years) was intentional, reflecting the diversity of adult music learners; age-related differences were examined in supplementary analyses.

### 2.3. Instruments

#### 2.3.1. Stroop Color-Word Test (Golden Version)

The Golden version of the Stroop Color-Word Test was administered according to standardized procedures [15,120]. The test comprises three 45 s trials: (1) Word Reading—reading color words (ΚΟΚΚΙΝΟ, ΠΡΑΣΙΝΟ, ΜΠΛΕ) printed in black ink; (2) Color Naming—naming the ink color of XXXX stimuli; and (3) Color-Word Interference—naming the ink color of incongruent color words. The Interference Score was calculated using Golden’s formula: Interference = Color-Word Score − [(Word Score × Color Score)/(Word Score + Color Score)]. The Greek adaptation has demonstrated adequate reliability and validity [121].

#### 2.3.2. Positive and Negative Affect Schedule (PANAS)

The PANAS [122,123] is a 20-item self-report measure yielding two subscales: Positive Affect (PA; 10 items: interested, excited, strong, enthusiastic, proud, alert, inspired, determined, attentive, active) and Negative Affect (NA; 10 items: distressed, upset, guilty, scared, hostile, irritable, ashamed, nervous, jittery, afraid). Participants rate each item on a 5-point scale (1 = very slightly or not at all; 5 = extremely). Subscale scores range from 10 to 50. The Greek validation [124] demonstrated excellent psychometric properties (PA: α = 0.85–0.90; NA: α = 0.84–0.87).

#### 2.3.3. State-Trait Anxiety Inventory—State Form (STAI-S)

The STAI-S [125,126] is a 20-item self-report scale measuring transitory emotional states characterized by subjective feelings of tension, apprehension, nervousness, and worry. Items are rated on a 4-point scale (1 = not at all; 4 = very much so). Total scores range from 20 to 80. Ten items are positively worded and reverse-scored. The Greek adaptation [127] demonstrated excellent reliability (α = 0.91–0.93) and sensitivity to acute changes in anxiety.

### 2.4. Procedure

Data collection occurred at participants’ regular music lesson locations. Upon arrival, participants provided informed consent and completed the pre-lesson assessment battery: (1) demographic questionnaire; (2) PANAS; (3) STAI-S; (4) Stroop test. Participants then attended their regular music lesson (45–60 min). Lessons followed standard pedagogical structures typical of conservatory-level instruction: (a) Warm-up phase (5–10 min): technical exercises, scales, or etudes; (b) Repertoire phase (25–35 min): work on assigned pieces, including interpretation, phrasing, and expression; (c) Review phase (10–15 min): feedback, goal-setting, and assignment of new material. Instructors (*N* = 12) were qualified music teachers with conservatory diplomas and minimum five years teaching experience. No specific emotional induction or standardized content was imposed; lessons reflected naturalistic music education practice, prioritizing ecological validity while acknowledging variability in lesson content.

### 2.5. Data Analysis

Prior to inferential analyses, distributional assumptions were evaluated. Normality was assessed using the Shapiro–Wilk test and visual inspection of Q-Q plots. Homogeneity of variance for between-group comparisons was verified using Levene’s test.

Data analysis was performed using Python 3.10 with packages scipy, statsmodels and semopy. The pre-post changes in data were analyzed through a series of statistical tests including Paired-Sample *t*-Tests which assessed the statistically significant changes in the group as well as the Cohen’s D effect size (i.e., small effect size d = 0.20, medium effect size d = 0.50, large effect size d = 0.80). Additionally, Pearson Correlations were used to assess the relationship between the change scores of the variables that were measured. The proposed model which links the affective changes to the cognitive improvements was assessed through multiple regression and path analysis. The model fit was also assessed using Chi-Square Index of Fit, Comparative Fit Index (CFI), Goodness-of-Fit Index (GFI) and Root Mean Square Error of Approximation (RMSEA). Mediation analysis followed Baron and Kenny’s procedure [40] with bootstrap confidence intervals (5000 resamples) for indirect effects, providing more robust inference than the Sobel test alone.

### 2.6. Ethical Considerations

Ethical review and approval were waived for this study, due to the University of Patras Ethics Committee and Research Ethics guidelines, as ethical approval is not required for studies involving anonymous survey-based research, mainly when the participants are healthy adults, not from vulnerable populations, and the study does not collect sensitive or identifiable personal data. Written informed consent was obtained from all adult participants prior to data collection. For participants under 18 years of age (*n* = 4), written assent was obtained from the participant, and written parental/guardian consent was obtained. Participants were informed of their right to withdraw at any time without consequence. All data were anonymized and stored securely in accordance with GDPR requirements.

## 3. Results

### 3.1. Preliminary Analyses

Shapiro–Wilk tests confirmed that all primary variables met normality assumptions: Stroop interference pre (W = 0.967, *p* = 0.112), Stroop interference post (W = 0.971, *p* = 0.158), positive affect pre (W = 0.963, *p* = 0.084), positive affect post (W = 0.969, *p* = 0.134), state anxiety pre (W = 0.954, *p* = 0.051), state anxiety post (W = 0.961, *p* = 0.071). All *p*-values exceeded 0.05, supporting the use of parametric statistics. Levene’s tests confirmed homogeneity of variance for between-group comparisons (all *p* > 0.10).

### 3.2. Participant Characteristics

Demographic characteristics are presented in Table 1. The sample comprised 60 musicians with balanced representation across instrument types.

### 3.3. Pre-Post Comparisons

Paired-samples *t*-tests revealed significant improvements across all measures (Table 2), supporting H1. Music lessons produced large improvements in Color Score (d = 1.46), Color-Word Score (d = 0.64), and Interference Score (d = 0.59). Positive affect increased substantially (d = 1.87), while negative affect (d = −2.34) and state anxiety (d = −2.64) showed large reductions.

**Figure 1:**
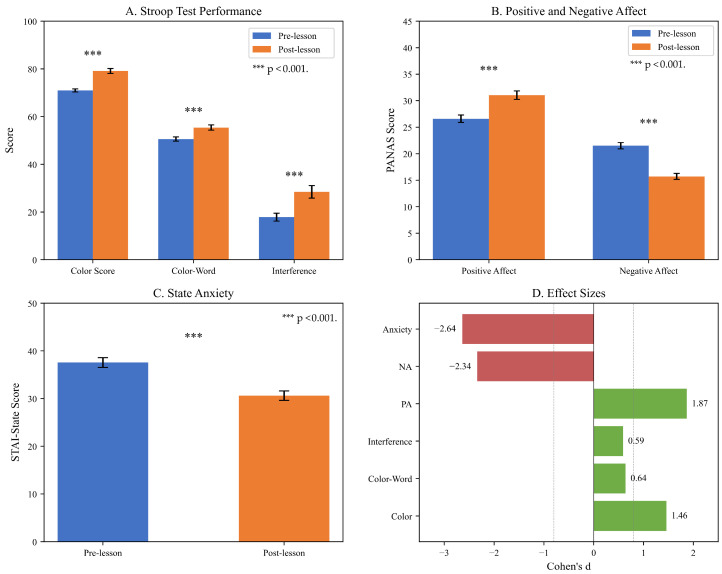
Pre-Post Comparison of Cognitive and Affective Measures. Note. PA = Positive Affect; NA = Negative Affect; STAI-S = State-Trait Anxiety Inventory-State subscale; d = Cohen’s d effect size. Error bars represent standard error of the mean. *** *p* < 0.001. (**Panel A**) shows improved performance on the Stroop test as measured by Color Score, Color-Word Score, and Interference Score. (**Panel B**) demonstrates increased Positive Affect (PA) and decreased Negative Affect (NA) on the PANAS scores. (**Panel C**) demonstrates a decrease in State Anxiety from the STAI. (**Panel D**) displays the effect sizes of each measure (Cohen’s d) with positive numbers indicating improvement (cognitive, PA) and negative numbers indicating a beneficial reduction (NA, anxiety). Standard error is represented by the error bars (*** *p* < 0.001).

To visualize the magnitude of changes across all measured domains, Figure 1 presents a four-panel comparison of Stroop performance, affective states, and corresponding effect sizes before and after the music lesson intervention.

### 3.4. Correlation Analysis

Correlation analysis revealed significant associations between affective changes and cognitive improvement (Table 3), partially supporting H2. Positive affect change correlated positively with interference score change (r = 0.564, *p* < 0.001), while anxiety change correlated negatively (r = −0.484, *p* < 0.001). However, negative affect change was not significantly correlated with cognitive improvement (r = −0.220, *p* = 0.091). Post-lesson interference scores correlated with negative affect (r = −0.260, *p* < 0.05) and age (r = 0.327, *p* < 0.05).

**Figure 2:**
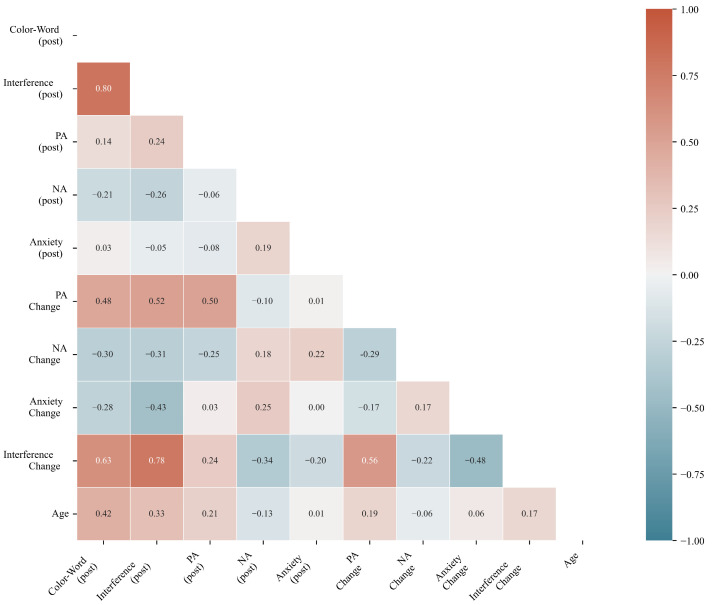
Correlation Heatmap of Post-Lesson Variables and Change Scores. Note. PA = Positive Affect; NA = Negative Affect. Values represent Pearson correlation coefficients (r).

The relationships among cognitive, affective, and demographic variables were examined through bivariate correlations. Figure 2 displays the complete correlation matrix, illustrating the strength and direction of associations between post-lesson measures and change scores.

Lower triangle displays Pearson correlation coefficients among cognitive measures (Color-Word, Interference), affective measures (PA, NA, Anxiety), change scores, and age. Significant correlations are highlighted (darker colors indicate stronger relationships). The pattern reveals distinct clusters: cognitive variables correlate strongly with each other (r = 0.80), and positive affect change shows the strongest association with cognitive improvement (r = 0.56).

### 3.5. Path Analysis

**Figure 3:**
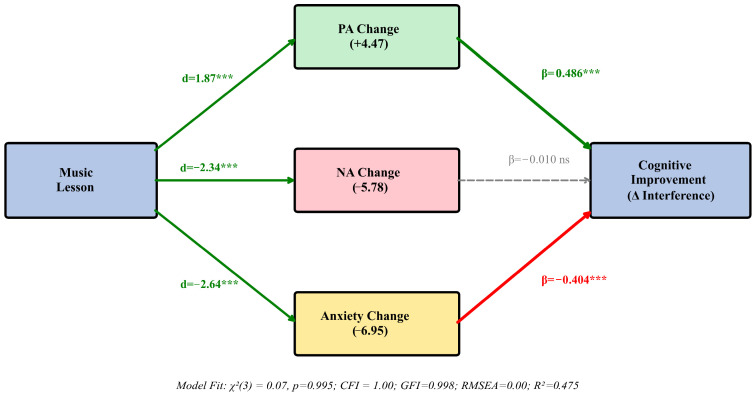
Path Analysis: Associations Between Affective Changes and Cognitive Improvement. Note. Standardized path coefficients (β) are displayed. CFI = Comparative Fit Index; RMSEA = Root Mean Square Error of Approximation; R^2^ = variance explained. PA = Positive Affect; NA = Negative Affect. *** *p* < 0.001; ns = not significant.

Path analysis examined the hypothesized model linking affective changes to cognitive improvement. The model demonstrated excellent fit: χ^2^(3) = 0.07, *p* = 0.995; CFI = 1.00; GFI = 0.998; RMSEA = 0.00. Results are presented in Table 4 and Figure 3.

Positive affect change (β = 0.486, *p* < 0.001) and anxiety change (β = −0.404, *p* < 0.001) were significantly associated with cognitive improvement, consistent with H3. Negative affect change was not a significant predictor (β = −0.010, *p* = 0.92). The model explained 47.5% of variance in interference score change.

To test the hypothesized affective mediation pathways, a path analysis model was constructed. Figure 3 presents the structural model with standardized path coefficients and model fit indices, depicting direct and indirect effects of music lessons on executive function.

Standardized path coefficients showing the dual pathway from music lessons to cognitive improvement through positive affect change (β = 0.486 ***) and anxiety reduction (β = −0.404 ***). Negative affect change shows no significant path (β = −0.010, ns). Green arrows indicate significant positive paths; red arrows indicate significant negative paths; dashed gray arrows indicate non-significant paths. Model demonstrates excellent fit indices.

### 3.6. Mediation Analysis

Mediation analysis tested whether positive affect change mediated the effect of music lessons on cognitive improvement. The total effect (c path) was significant (d = 0.59, *p* < 0.001). The effect of music lessons on positive affect (a path) was large (d = 1.87, *p* < 0.001). The effect of positive affect change on cognitive improvement controlling for the lesson effect (b path) was significant (r = 0.56, *p* < 0.001).

The indirect effect (a × b = 1.05) was significant as confirmed by the Sobel test (z = 4.89, *p* < 0.001). The proportion of the total effect mediated by positive affect was 38.2%. These results are consistent with partial mediation, suggesting that the association between music lessons and cognitive performance may operate both directly and through concurrent increases in positive affect. However, the single-group design limits causal interpretation of these pathways.

**Figure 4:**
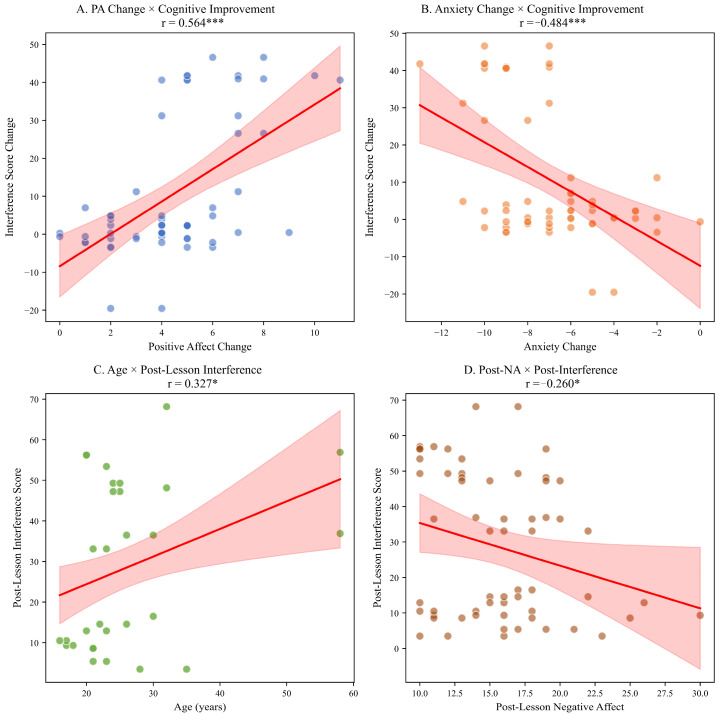
Scatter Plots with Regression Lines for Key Relationships. Note. PA = Positive Affect; NA = Negative Affect. Shaded areas represent 95% confidence intervals. * *p* < 0.05; *** *p* < 0.001. (**Panel A**): Positive affect change vs. interference score change (r = 0.564 ***). (**Panel B**): Anxiety change vs. interference score change (r = −0.484 ***). (**Panel C**): Age vs. post-lesson interference score (r = 0.327 *). (**Panel D**): Post-lesson negative affect vs. post-lesson interference score (r = −0.260 *). Shaded areas represent 95% confidence intervals.

The nature of key relationships was further explored through regression analysis. Figure 4 presents scatter plots illustrating the associations between positive affect and cognitive improvement, anxiety and cognitive change and the moderating influences of age and negative affect.

**Figure 5:**
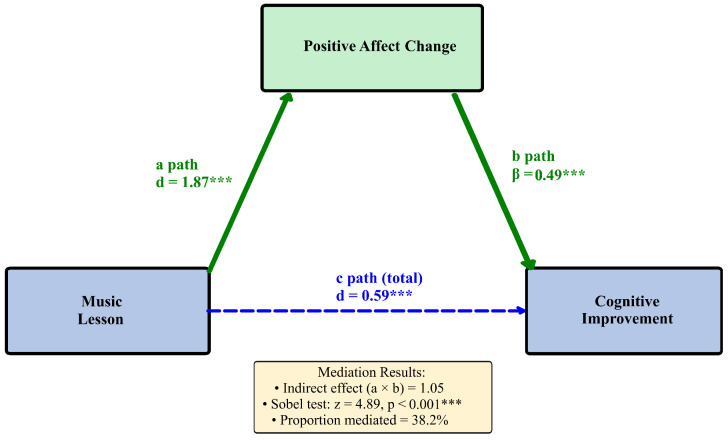
Statistical Mediation Model: Positive Affect as Potential Pathway. Note. Path a = effect of music lesson on mediator; Path b = effect of mediator on cognitive outcome; Path c = total effect. *** *p* < 0.001.

The complete mediation analysis, testing whether affective changes account for the relationship between music lessons and cognitive enhancement, is depicted in Figure 5. The diagram presents the a, b, c, and c’ paths with bootstrap confidence intervals for indirect effects.

Path diagram showing the mediation of music lesson effects on cognitive improvement through positive affect change. The a path (d = 1.87 ***) represents the lesson effect on positive affect; the b path (β = 0.49 ***) represents the positive affect effect on cognition; the c path represents the total effect; c’ represents the direct effect after controlling for the mediator. Indirect effect = 1.05, Sobel z = 4.89 ***. Proportion mediated = 38.2%.

### 3.7. Supplementary Analysis: Age-Controlled Models

Given the significant correlation between age and cognitive change (r = 0.287, *p* = 0.026), supplementary path analyses were conducted with age as a covariate. In the age-controlled model, the associations between positive affect change and cognitive improvement (β = 0.46, *p* < 0.001) and between anxiety change and cognitive improvement (β = −0.38, *p* < 0.001) remained significant with overlapping confidence intervals compared to the unadjusted model. These results suggest that the observed associations are not attributable to age-related confounding.

### 3.8. Gender Differences

Independent *t*-tests revealed significant gender differences in post-lesson interference scores (Table 5). Females demonstrated higher interference scores (M = 34.13, SD = 20.19) than males (M = 21.06, SD = 18.46), t(58) = −2.58, *p* = 0.013, d = 0.68. No significant gender differences were found for affective measures.

**Figure 6:**
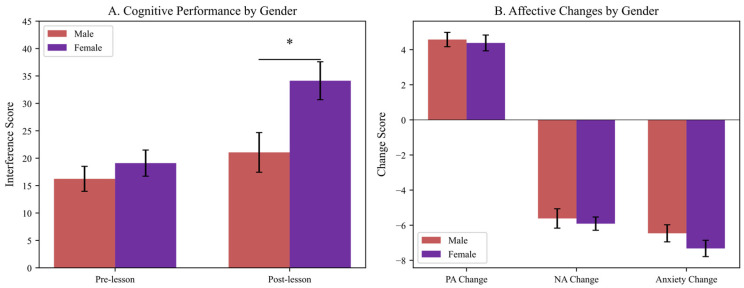
Gender Differences in Cognitive and Affective Outcomes. Note. PA = Positive Affect; NA = Negative Affect. Error bars represent standard error of the mean. * *p* < 0.05. (**Panel A**): Executive function (interference scores) by gender, showing pre- and post-lesson means. Females demonstrated significantly higher post-lesson interference scores than males (*p* = 0.013, d = 0.68). (**Panel B**): Affective changes (PA, NA, anxiety) by gender, showing no significant differences. Error bars represent standard errors.

Gender differences in response to music education were examined across all outcome measures. Figure 6 illustrates executive function and affective changes by gender, with females demonstrating greater cognitive improvement compared to males.

### 3.9. Instrument Differences

One-way ANOVA revealed a trend toward significant differences in interference scores across instruments, F(2, 57) = 3.04, *p* = 0.055, η^2^ = 0.097. Violinists showed the highest post-lesson interference scores (M = 37.11, SD = 17.32), followed by pianists (M = 25.90, SD = 21.75) and guitarists (M = 22.39, SD = 19.79). No significant instrument differences were found for affective measures (all ps > 0.27).

**Figure 7:**
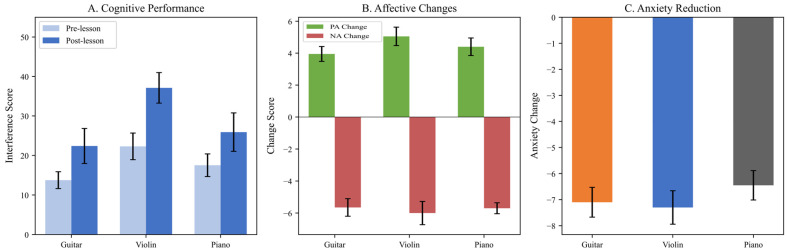
Instrument Comparison: Cognitive and Affective Outcomes by Instrument Type. Note. PA = Positive Affect; NA = Negative Affect. Error bars represent standard error of the mean. (**Panel A**): Cognitive inhibition (interference scores) showing pre- and post-lesson means for guitar, violin, and piano groups. Violinists demonstrated the highest post-lesson scores (trend-level significance, *p* = 0.055). (**Panel B**): Affective changes by instrument, showing similar patterns of positive affect increase and negative affect decrease across all instruments. (**Panel C**): Anxiety reduction by instrument. Error bars represent standard errors.

To examine whether cognitive and affective benefits varied by musical instrument, between-group comparisons were conducted. Figure 7 displays the cognitive and affective outcomes across guitar, violin, and piano groups, revealing instrument-specific patterns of enhancement.

### 3.10. Distribution Analysis

**Figure 8:**
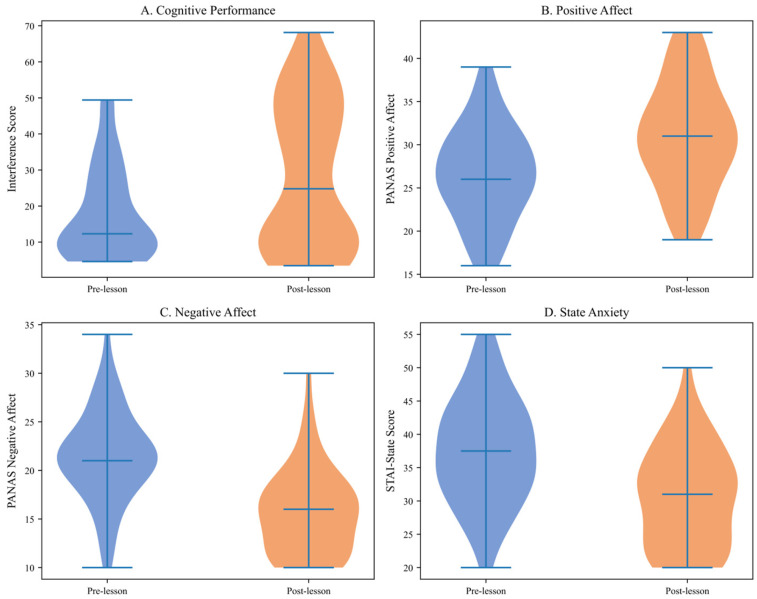
Distribution of Pre- and Post-Lesson Scores (Violin Plots). Note. STAI-S = State-Trait Anxiety Inventory-State subscale. Thick bars indicate interquartile ranges. (**Panel A**): Interference score distributions showing rightward shift post-lesson, indicating improved cognitive control. (**Panel B**): Positive affect distributions showing upward shift. (**Panel C**): Negative affect distributions showing downward shift. (**Panel D**): State anxiety distributions showing marked reduction. White dots indicate medians; thick bars indicate interquartile ranges. The visualizations demonstrate consistent improvements across all participants with minimal floor/ceiling effects.

To illustrate the distributional characteristics of score changes, Figure 8 presents violin plots displaying the full distribution of pre- and post-lesson scores across all measures, revealing both central tendencies and individual variability in response to the intervention.

## 4. Discussion

The overall objective of this study was to examine the acute cognitive and affective effects of single music lessons in trained musicians and to explore associations between affective changes and cognitive outcomes. The results provide support for all hypotheses: executive function improved significantly following the lesson (H1), affective states changed in the predicted directions (H2 supported), affective and cognitive changes were significantly correlated (H2 supported), and statistical mediation models indicated that affective variables were associated with the lesson-cognition relationship (H3 supported). These findings are discussed below in relation to existing literature, with careful attention to the limitations imposed by the single-group pre-post design.

The results from this study provide new insight into the psychological factors through which music can improve cognitive functions. Using executive function, emotional states, and anxiety levels before and after taking music lessons, we found that music education can improve cognition and emotion; also, that some portion of these cognitive enhancements may be related to the emotional state changes caused by the experience. The data obtained support the growing body of knowledge on music’s potential role as a multidimensional treatment modality that engages multiple interconnected brain systems that regulate emotions and high-level cognitive processing.

### 4.1. Principal Findings

Significant improvements in executive function (measured as Stroop interference) occurred following music lessons, resulting in a large increase in positive affect and a large decrease in negative affect and state anxiety. It was also noted that the degree of change in positive and negative affect was larger than that observed in cognitive changes; therefore, it appears that music engagement has a greater influence on emotional processing systems. These findings corroborate prior studies on mood elevation following musical activity and provide additional insight into how emotional changes may be related to cognitive outcomes.

The relationship between affective and cognitive changes observed in this study exhibited a complex, theoretically relevant pattern. Both positive affect enhancements and anxiety decreases were significantly correlated with cognitive improvements. However, the relationship between executive function gains and changes in negative affect was not statistically significant. This finding supports the idea that the benefits of music lessons occur via at least two specific pathways: the activation of positive emotional states and the reduction in anxiety, rather than occurring through a general reduction in negative mood. A neuroscientific interpretation of these findings is consistent with prior research indicating that positive affect and anxiety elicit activation of distinct brain regions, each with distinct connectivity to prefrontal areas responsible for executive function (e.g., [21,22]). In addition, the anterior cingulate cortex and dorsolateral prefrontal cortex, which are critical for successful completion of Stroop tasks [56,57], receive modulatory input from both limbic regions responsible for reward and those responsible for threat. Therefore, a dual pathway model, similar to the one described above, can be supported at a neuroanatomical level.

A path analysis indicated that both positive affect change and anxiety reduction independently predicted cognitive improvement and accounted for a significant amount of variance in executive function gains. Furthermore, mediation analysis demonstrated that positive affect partially mediated the relationship between music lesson participation and cognitive improvement, thereby supporting the notion that a substantial amount of cognitive benefit results from an increased positive emotional response to learning experiences. These findings provide empirical support for incorporating affective measures into the investigation of cognitive enhancement and indicate that emotional responses to learning experiences are not simply epiphenomenal but contribute to the cognitive benefits derived from those experiences.

### 4.2. Theoretical Implications

These findings contribute to the theoretical understanding of how music influences cognition, provide evidence for the specific mechanisms of affective influence (the processes by which music produces its effects), and position these mechanisms relative to other theoretical frameworks developed in cognitive neuroscience. The significant mediation of the relationship between music-induced positive emotion and reduced interference error provides support for Fredrickson’s “broaden and build” theory [65] and her notion that positive emotion expands one’s scope of attention and cognition and builds enduring personal resources. Music-induced positive affect may broaden attentional scope or cognitive flexibility, allowing individuals to resolve the conflict that arises in response to the Stroop Task more effectively.

The independent contributions of anxiety reduction demonstrate a relationship consistent with Eysenck’s processing efficiency theory [75,76], in which Eysenck argued that anxiety impairs executive function (including the ability to perform tasks requiring executive functions) by consuming working memory resources through worry and threat monitoring. Therefore, if music lessons reduce an individual’s state anxiety, they may free up cognitive resources consumed by worry and threat monitoring, thereby improving performance on executive function tasks that require a high degree of executive functioning.

This dual pathway model—the pathway of positive affect enhancement and the pathway of anxiety reduction—represents a theoretical contribution with implications that extend far beyond music education. This model indicates that programs designed to enhance cognitive abilities will likely be more effective if they also include components that cultivate positive emotional states and manage anxiety, whereas it seems unlikely that this would occur if the program focused only on cognitive training. As noted above, there is growing recognition in the field of neuroplasticity that emotional states impact the processes of learning and memory consolidation, including the fact that positive affect enhances encoding, while anxiety reduces retrieval and impairs executive functioning [42,43]. Due to music’s unique ability to engage both emotional and cognitive processes simultaneously, music education appears to be an optimal way to leverage the brain-based learning principles mentioned above.

Finally, the cultural embeddedness of musical practice contributes additional theoretical complexity. Music is not simply an auditory stimulus, but rather a culturally meaningful activity that engages social, emotional, and identity-related processes. Thus, it is possible that the cognitive benefits reported in this study were, at least in part, due to the cultural importance of musical participation for participants. Therefore, it may be expected that similar interventions may have different effects depending upon cultural context, musical tradition, and value systems [101]. Future theoretical work should incorporate cultural neuroscience’s views on the bidirectional relationship between cultural practices and brain function [102,103,104].

However, the present correlational design cannot establish that affective changes causally underlie cognitive improvement. The observed statistical mediation reflects patterns of covariation consistent with theoretical predictions but requires experimental verification through designs that manipulate affective states independently of music engagement.

### 4.3. Practical Implications

The following practical implications are offered cautiously, recognizing that the single-group pre-post design limits causal inference. The significance of these findings is that they provide a basis for building educational practices and music pedagogy, and for implementing music-based interventions to support cognitive and emotional well-being. In addition, in the context of educational neuroscience, this study demonstrates that music education is an example of a brain-based intervention that targets both cognitive and affective systems (an approach to teaching and learning increasingly recognized as required for effective learning) [44,84]. Therefore, the results of this study provide compelling evidence to support the use of music lessons to develop students as a whole; the findings also indicate that music lessons can have a very large impact on the cognitive functioning of students, as well as their emotional state, and therefore could provide a benefit to students who experience anxiety or have difficulty regulating their own emotional states—many of whom will experience difficulty achieving academically. Systems of education seeking evidence-based approaches to support students who are struggling to learn might find music education a useful tool when combined with other forms of support to implement a comprehensive approach.

Furthermore, the mediation findings demonstrate several practical ways to enhance the cognitive aspects of music education. Since positive effects have been shown to mediate cognitive enhancement, music education instructional methods that create a positive environment and promote positive emotional engagement may produce greater cognitive enhancements than those that focus primarily on technical skills. Therefore, music educators may want to incorporate methods into their teaching practices that increase positive experiences (e.g., supportive feedback, appropriately challenging goals, personally meaningful repertoire, and collaborative music making) while decreasing anxiety-producing variables (e.g., high performance expectations, negative evaluations).

Additionally, the gender difference found in this study (female participants demonstrated greater cognitive improvements) should also be considered within the framework of educational practice. These differences may arise from differences in emotional connection, learning preferences, and socialization processes associated with involvement in music experiences. Individualized approaches to music education are essential for developing effective programs that can be adapted to meet the needs of diverse learners. The use of emerging AI technologies presents an opportunity to adapt music education to each learner’s unique cognitive/affective profile. In doing so, this may help alleviate the problems of gender and individual differences through the use of a personalized intervention [95,99].

In addition, the trend-level differences across the various types of instruments used in music education (i.e., string players demonstrated higher cognitive scores) raise interesting questions about the potential differential cognitive demands of these instruments. Each type of instrument engages multiple motor systems, creates varying levels of bimanual coordination, and produces varying relationships between movement and auditory feedback. Studies of the effects of instrumentation may also inform educators which instrument(s) would be most beneficial for achieving specific cognitive or therapeutic goals. Using deep learning techniques to analyze multimodal data from music learning environments may allow researchers to better understand what features of musical engagement are most closely linked to cognitive benefits [54,77].

Lastly, the findings of this study provide a basis for expanding the use of music therapy and clinical applications. The significant decrease in anxiety observed in this study indicates that engaging in music may serve as an effective means of reducing anxiety in educational, clinical, and occupational settings. Additionally, the finding that reductions in anxiety contribute independently to cognitive improvements supports the rationale for developing music-based interventions for anxiety and suggests that such interventions may produce cognitive as well as emotional benefits [58,59,60,61,62,63]. Using physiological monitoring and delivering music in real time—an application now possible due to advancements in wearable technology and AI—may enable the creation of precision music therapy interventions tailored to each learner’s psychological and physiological state [24,77,99].

However, generalization from the present single-session, single-group study to policy recommendations or clinical applications would be premature. Randomized controlled trials with appropriate comparison conditions are necessary before implementing music-based interventions based on these findings.

### 4.4. Limitations and Future Directions

As a pilot study, these findings are exploratory in nature and cannot be generalized to broader populations. The absence of a control group and randomization precludes causal inference. The present results are intended to generate preliminary evidence and inform the design of future controlled studies with larger, more representative samples. Several limitations warrant careful consideration. Most critically, the single-group pre-post design without a control condition precludes causal inference. Observed changes may reflect practice effects from repeated Stroop administration, regression to the mean, demand characteristics, expectancy effects, or temporal factors unrelated to the music lesson itself.

While acknowledging several methodological concerns and identifying areas that hold promise for the future of research into this topic, the use of a within-subjects pre-post experimental design provides sufficient power to demonstrate acute changes but does not allow for establishing causation. It is possible that some degree of improvement can be attributed to practice effects, expectancy effects, or normal fluctuations in the temporal dimension. Future randomized controlled trials using active control conditions will provide stronger evidence regarding the extent to which music-engagement contributes to cognitive enhancements.

Regarding sample characteristics, the results presented here cannot be generalized. The participants were Greek musicians who were actively engaged in instrumental training. As such, it raises questions regarding the ability to generalize the findings across different cultural contexts. Since music education is a culturally bound construct, it is necessary to conduct cross-cultural investigations to understand how socio-cultural factors influence the affective-cognitive pathways identified in this study.

As we move forward with research on multisensory learning, it is increasingly apparent that the mechanisms of neuroplasticity underlying learning can be culturally/linguistically driven. The implication is that the cognitive improvements associated with music training are likely to vary significantly across cultures/populations [101,105,106,107,108,109,110,111]. Further comparative research involving musicians from various cultural backgrounds will help determine whether the cognitive improvements resulting from music training are universal or culturally specific.

In addition to the cultural/linguistic implications, methodologically, the use of self-report measures of affective states has several limitations, including potential biases in reporting subjective experiences. Recent advancements in objective neuroimaging technologies offer promising methods for assessing subjective emotional and cognitive processes. One such method is electroencephalography (EEG), which has demonstrated sensitivity to music-induced affective states [24,25]. As an alternative to the subjectivity of self-reporting, EEG could also be used to investigate the real-time neurophysiological correlations of the affective changes reported in this study. The integration of EEG-based emotion recognition with behavioral measures would enable researchers to more accurately characterize the temporal relationships among musical engagement, affective state, and cognitive performance. The mapping of EEG metrics to existing affective and cognitive models provides researchers with a framework to interpret neurophysiological data in relation to psychologically meaningful constructs [25].

While the current study examined the acute effects of a single music lesson, the impact of long-term music training on cognitive-affective development remains a major unknown. Therefore, additional longitudinal research tracking students across multiple years will provide insight into the developmental trajectories and neuroplastic changes associated with music education. These studies could build upon recent developments in educational neuroscience, highlighting the dynamic relationship between experience and neural plasticity [44]. In addition, understanding how repeated exposure to music affects brain structure and function over time presents a significant area of growth for the field [45,46,47,48,49,50,51].

Finally, the use of artificial intelligence (AI), adaptive assessment technology, and personalized learning systems represents a promising avenue for advancing the science of music education. For example, AI systems that are based on the principles of cognitive neuropsychology could adapt music instruction to the individual needs of each student using their cognitive learner profile [95]. Furthermore, deep-learning algorithms capable of identifying emotional states from multimodal data, including facial expressions, physiological signals, and behavioral responses, could enable real-time adjustments to musical activities to maximize both affective and cognitive outcomes [54,77]. Finally, the concept of digital twin cognition, in which AI systems represent individual cognitive/emotional profiles, provides a framework for developing precision interventions in music education tailored to individual differences in learning [99]. Importantly, contemporary theoretical perspectives emphasize that effective learning requires more than traditional cognitive load management, highlighting the critical role of affective and motivational processes in optimizing educational outcomes [129]. Such technological advancements have the potential to transform music education from a traditional, standardized model to a customizable, cognitive/emotional-optimization model.

## 5. Conclusions

This pilot study examined the acute effects of single music lessons on cognitive and affective outcomes in trained adult musicians. Executive function improved significantly following music lessons, indicating enhanced inhibitory control after musical engagement. Positive affect increased substantially, while negative affect and state anxiety decreased, suggesting that single music lessons are associated with meaningful affective shifts. Changes in positive affect and state anxiety were significantly correlated with cognitive improvement, indicating that participants who experienced greater affective benefits also showed greater cognitive gains. Statistical mediation analyses indicated that affective changes were associated with a substantial proportion of the relationship between lesson participation and cognitive change, consistent with theoretical models positing affective mechanisms in music-cognition relationships. Limitations: As a pilot study, these findings are exploratory and cannot be generalized to broader populations. The single-group pre-post design does not permit causal conclusions. The observed associations may reflect practice effects, demand characteristics, or temporal confounds rather than true intervention effects. Replication with randomized controlled designs and larger samples is necessary before practical applications can be recommended. Future Directions: Controlled trials with active comparison conditions, neuroimaging measures, and longitudinal follow-up are needed to test causal hypotheses and evaluate the durability of effects. Cross-cultural replication would enhance the generalizability of findings. In summary, this pilot study provides preliminary evidence that single music lessons are associated with both cognitive and affective benefits in trained musicians, with affective changes statistically linked to cognitive outcomes. These exploratory findings warrant further investigation using designs capable of supporting causal inference and generalization.

## Figures and Tables

**Table 1:** Demographic Characteristics (*N* = 60).

Variable	*n*/M	%/SD
Gender		
Male	26	43.3%
Female	34	56.7%
Age (years)	26.0	9.8
Range	16–58	
Instrument		
Guitar	20	33.3%
Violin	20	33.3%
Piano	20	33.3%

Note. *N* = 60; *n* = number of participants; M = mean; SD = standard deviation; % = percentage.

**Table 2:** Pre-Post Paired *t*-Tests (*N* = 60).

Variable	Pre M (SD)	Post M (SD)	t (59)	*p*	d
Color Score	70.97 (5.09)	79.13 (7.95)	11.33	<0.001	1.46
Color-Word	50.57 (6.79)	55.40 (8.47)	4.96	<0.001	0.64
Interference	17.86 (12.94)	28.47 (20.38)	4.55	<0.001	0.59
Positive Affect	26.58 (5.44)	31.05 (6.23)	14.48	<0.001	1.87
Negative Affect	21.50 (4.64)	15.72 (4.41)	−18.13	<0.001	−2.34
State Anxiety	37.57 (8.01)	30.62 (7.56)	−20.45	<0.001	−2.64

Note. *N* = 60; M = mean; SD = standard deviation; t = *t*-test statistic; *p* = *p*-value; d = Cohen’s d effect size. Significance levels: *p* < 0.001 (high significance), *p* < 0.01 (medium significance), *p* < 0.05 (low significance). Effect size interpretation [128]: |d| < 0.20 (no effect), |d| = 0.20–0.49 (small effect), |d| = 0.50–0.79 (moderate effect), |d| ≥ 0.80 (large effect).

**Table 3:** Correlations Among Change Scores and Key Variables.

Variable	1	2	3	4	5
1. Interference Δ	—				
2. PA Change	0.564 ***	—			
3. NA Change	−0.220	−0.312 *	—		
4. Anxiety Change	−0.484 ***	−0.178	0.181	—	
5. Age	0.287 *	0.156	−0.089	−0.045	—

Note. *N* = 60; PA = Positive Affect; NA = Negative Affect; Δ = change score (post minus pre). Significance levels: * *p* < 0.05 (low significance), *** *p* < 0.001 (high significance).

**Table 4:** Path Analysis Results: Predictors of Cognitive Improvement.

Predictor	B	SE	β	z	*p*
PA Change	3.74	0.77	0.486	5.15	<0.001 ***
NA Change	−0.07	0.75	−0.010	−0.10	0.920
Anxiety Change	−2.74	0.68	−0.404	−4.29	<0.001 ***

Note. *N* = 60; B = unstandardized coefficient; SE = standard error; β = standardized coefficient; z = z-statistic; *p* = *p*-value. PA = Positive Affect; NA = Negative Affect. Model fit indices: Significance levels: *** *p* < 0.001 (high significance).

**Table 5:** Gender Differences in Post-Lesson Measures.

Variable	Male M (SD)	Female M (SD)	t	*p*	d
Interference	21.06 (18.46)	34.13 (20.19)	−2.58	0.013 *	0.68
Positive Affect	30.42 (6.18)	31.53 (6.32)	−0.68	0.500	0.18
Negative Affect	16.15 (5.02)	15.38 (3.92)	0.67	0.506	0.17
State Anxiety	31.27 (7.36)	30.12 (7.78)	0.58	0.563	0.15

Note. *N* = 60 (Male *n* = 26; Female *n* = 34); M = mean; SD = standard deviation; t = *t*-test statistic; *p* = *p*-value; d = Cohen’s d effect size. Significance levels: * *p* < 0.05 (low significance). Effect size interpretation [128]: |d| < 0.20 (no effect), |d| = 0.20–0.49 (small effect), |d| = 0.50–0.79 (moderate effect), |d| ≥ 0.80 (large effect).

## Data Availability

The raw data supporting the conclusions of this article will be made available by the authors on request.

## Abbreviations

The following abbreviations are used in this manuscript:

ACC	Anterior Cingulate Cortex
AI	Artificial Intelligence
C	Color Score
CFI	Comparative Fit Index
CW	Color-Word Score
DLPFC	Dorsolateral Prefrontal Cortex
EEG	Electroencephalography
EF	Executive Function
fMRI	Functional Magnetic Resonance Imaging
GFI	Goodness of Fit Index
NA	Negative Affect
PA	Positive Affect
PANAS	Positive and Negative Affect Schedule
PFC	Prefrontal Cortex
RMSEA	Root Mean Square Error of Approximation
SEM	Structural Equation Modeling
STAI	State-Trait Anxiety Inventory
STAI-S	State-Trait Anxiety Inventory-State Subscale
W	Word Score
